# Liquid Core ARROW Waveguides: A Promising Photonic Structure for Integrated Optofluidic Microsensors

**DOI:** 10.3390/mi7030047

**Published:** 2016-03-11

**Authors:** Genni Testa, Gianluca Persichetti, Romeo Bernini

**Affiliations:** Istituto per il Rilevamento Elettromagnetico dell’Ambiente, Consiglio Nazionale delle Ricerche (IREA-CNR), Via Diocleziano 328, 80124 Naples, Italy; persichetti.g@irea.cnr.it (G.P.); bernini.r@irea.cnr.it (R.B.)

**Keywords:** liquid ARROW, optofluidics, microfluidics, optical sensors, integrated silicon technology

## Abstract

In this paper, we introduce a liquid core antiresonant reflecting optical waveguide (ARROW) as a novel optofluidic device that can be used to create innovative and highly functional microsensors. Liquid core ARROWs, with their dual ability to guide the light and the fluids in the same microchannel, have shown great potential as an optofluidic tool for quantitative spectroscopic analysis. ARROWs feature a planar architecture and, hence, are particularly attractive for chip scale integrated system. Step by step, several improvements have been made in recent years towards the implementation of these waveguides in a complete on-chip system for highly-sensitive detection down to the single molecule level. We review applications of liquid ARROWs for fluids sensing and discuss recent results and trends in the developments and applications of liquid ARROW in biomedical and biochemical research. The results outlined show that the strong light matter interaction occurring in the optofluidic channel of an ARROW and the versatility offered by the fabrication methods makes these waveguides a very promising building block for optofluidic sensor development.

## 1. Introduction

Optofluidics is an emerging research field that combines the advantages of microfluidics and optics on the same platform towards highly functional and compact devices [1,2]. More precisely, optofluidic approaches provide high-yield integration of fluidics by including fluidic elements as parts of the photonic structure. This solution confers to the optofluidic systems a high level of compactness and reconfigurability as the fluids provide an effective means to tune and reconfigure the photonic architecture.

In the field of optical sensor technologies, optofluidics offers innovative design solutions in order to integrate all the required microfluidic and optical functionalities on a single chip for high-throughput analysis. Optofluidic sensors have been extensively developed for biomedical and biochemical analysis for healthcare purposes (*i.e.*, diagnostic), pharmaceutical research, and environmental monitoring [3]. Whatever optical method is used, highly-sensitive detection requires a very careful design of both optical and microfluidic parts in order to push the device sensitivity to the limit of the theoretical capability. Furthermore, innovative design strategies need to be developed to overcome the reduced interaction length owing to devices size miniaturization. In this context, optofluidics answer to the growing need for miniaturized and portable sensing devices, highly integrated, and extremely sensitive [4].

Optofluidic waveguides represent one of the simplest examples of optofluidic integration, as the optical and fluidic structures are tightly integrated into each other. In particular, an optofluidic waveguide is a photonic structure able to perform optical confinement of the light through a fluid [5]. Among others, liquid cores antiresonant reflecting optical waveguides (ARROWs) have been revealed as a very powerful building-block for optofluidic sensors [6]. In an ARROW the light is guided through the liquid core by means of highly reflective mirrors suitably realized on the core sidewalls. Since both the light and the liquid are guided through the same microchannel, ultra-high sensitivity can be achieved by exploiting the direct interaction of the entire optical waveguide mode with the fluid samples. Such a strong optical coupling, combined with the ultra-small liquid volume, enables sensitivity down to single molecule level, which is the ultimate goal of analytical methods. ARROWs can be easily fabricated using silicon technologies; moreover, like others optofluidic waveguides, such as the photonic crystal [7,8] and the slot waveguides [9,10], ARROWs are, in essence, planar and, hence, highly attractive for planar optofluidic integration [11,12]. As a matter of fact, starting from the first demonstration of the ARROWs’ potentialities in the field of optical sensing, there has been a growing interest for them as basic elements in optofluidic chip for sensing applications.

In the following, we report on optofluidic sensors realized with liquid ARROWs. Starting from the description of the working principle and fabrication methods of liquid ARROWs, we review the state of the art of on chip integration for sensing applications, highlighting specific advantages gained through the optofluidic integration.

## 2. Operation Principles and Simulations

### 2.1. 1-D Liquid ARROW

**Figure 1:**
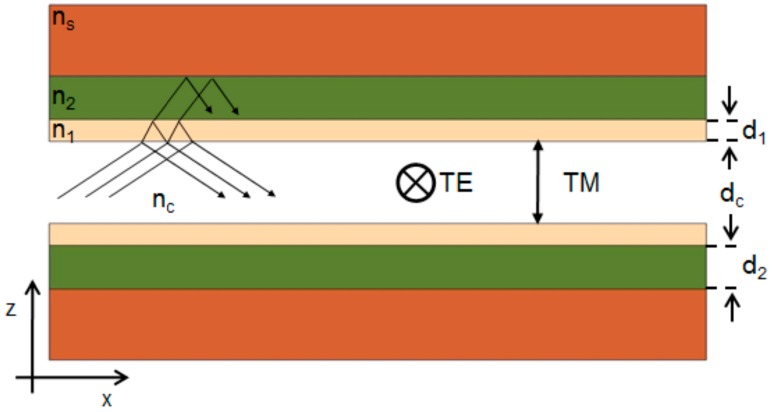
1-D structure of ARROW waveguide.

ARROW waveguides enable light confinement and propagation thanks to interference phenomena occurring in the cladding structure, made up of alternating high- and low- refractive index (RI) thin layers. The working principle of an ARROW waveguide can be easier understood by considering a schematic one-dimensional structure, as shown in Figure 1. For simplicity, we consider only two cladding layers with refractive index *n*_1_ and *n*_2_ (*n*_1_ > *n*_2_). Light propagates through the core by means of Fresnel reflections at the cladding interfaces. The entrapped light in each cladding layer experiences interference due to repeated reflections at cladding interfaces. In order to intensify the light reflected back into the core, each interference cladding must be designed to fulfill a specific condition. By considering the analogy of a cladding layer with a Fabry–Pérot cavity, such condition corresponds to the anti-resonance state of the cavity, which performs the maximum reflectivity. The reflectivity of the multilayer stack constituted by multiple interference claddings, generally called Fabry–Pérot mirror, can be very high, up to 99%.

A detailed description of the ARROW confinement phenomenon is reported in [13], where the phase accumulated by the fundamental mode at each round trip in the interference claddings is clearly defined. By setting the specific condition of antiresonance, a very useful expression to calculate the optimal cladding thicknesses *d*_1_ and *d*_2_, can be derived:

(1) $$d_{1,2}=\frac{\lambda }{4}{\left[n_{1,2}^{2}-n_{eff}^{2}\right]}^{\frac{-1}{2}}\left(2N+1\right)\;N=0,\;1,\;2,\;\ldots$$

where λ is the working wavelength, *n*_1,2_ is the refractive index of the cladding layers and *n_eff_* is the effective index of the propagation mode. For the fundamental mode, *n_eff_* can be approximately given by:

(2) $$n_{eff}=\frac{\beta }{\beta_{0}}≅n_{c}{\left[1-{\left(\frac{\lambda }{2n_{c}d_{c}}\right)}^{2}\right]}^{\;\frac{1}{2}}$$

where β_0_ = 2π/λ, β is the propagation constant, *n_c_* and *d_c_* are the refractive index and the thickness of the core, respectively. If λ/*d_c_* << 1 (e.g., large core waveguides), Equation (2) can be simplified to *n_eff_* ≈ *n_c_*. As long as the operating wavelength and the core and cladding materials are conveniently chosen, reduced propagation losses can be obtained by using Equations (1) and (2) to calculate the cladding thickness.

It should be noted that, unlike photonic crystal waveguides, in ARROW waveguides the high- and low-index alternating layers do not require a high periodicity in the structure [14]. This characteristic simplifies the waveguides manufacturing and strongly relaxes the fabrication constrains.

ARROWs are leaky waveguides and require a very careful design to operate in a single-mode condition, which is a fundamental requirement for sensing applications, *i.e.*, for the fabrication of interferometric devices. Single-mode operation can be accomplished by exploiting the weaker reflectivity experienced by higher order modes upon reflections at cladding interfaces. Low-order modes propagate with glancing angles at cladding interfaces, hence the Fresnel coefficient is quite high and the loss is low, whereas higher-order modes have larger incident angles and hence experience higher losses. An extensive investigation of the ARROW attenuation losses has been reported in [15]. The authors provide very useful analytical expressions that, with a good approximation, describe the mode losses for both TE and TM-polarized modes. A simple calculation form analytical expression given in [15] permits to calculate the ARROW attenuation coefficient for the TE and TM fundamental modes in the following, very useful, approximated form:

(3) $$\left\{ \begin{array}{l} \alpha_{TE}=\frac{0.5\lambda^{4}\left[1+\frac{4d_{c}^{2}}{\lambda^{2}}\left(n_{2}^{2}-n_{c}^{2}\right)\right]}{n_{c}d_{c}^{5}\left(n_{1}^{2}-n_{c}^{2}\right)\sqrt{\left(n_{s}^{2}-n_{c}^{2}\right)}}\\ \alpha_{TM}={\left(\frac{n_{1}^{2}n_{s}}{n_{c}n_{2}^{2}}\right)}^{2}\alpha_{TE} \end{array}\right.$$

which is valid when *d_c_* >> λ; *n_s_* is the refractive index of the substrate. By assuming Si_3_N_4_ (*n*_1_ = 2.01) and SiO_2_ (*n*_2_ = 1.44) for the first and second layer, Si (*n_s_* = 3.8) for the substrate, and water core (*n_c_* = 1.33), the proportional coefficient between α_TM_ and α_TE_ in Equation (3) is as high as 30. Hence the propagating modes experience attenuation coefficients that strongly depend upon their state of polarization. In particular, propagation of TE-polarized light is always privileged due to the lower attenuation coefficients. Additionally, in this case this behavior originates from the light confinement mechanism, based on Fresnel reflections at core and cladding boundaries. Fresnel reflection depends on the polarization of the incident light and TM reflection coefficient is always lower with respect to TE reflection.

Moreover, as a general rule, from Equation (3) the attenuation coefficients decrease by increasing the core dimensions. This implies that the core size must be suitably chosen in order to reduce fundamental mode loss but also to minimize the contribution of higher order modes to the total transmitted power. The degree of suppression of these modes improves as the core width decreases [16]. A further very useful general rule can be retrieved: in order to reduce the attenuation coefficients, the refractive index of the first cladding (*n*_1_) should be higher than the core index, while the refractive index of the second cladding (*n*_2_) as closer as possible to the core index (*n_c_*). These requirements impose very important limitations to the choice of the cladding materials. For this reason typical materials are: silicon nitride (*n*_1_ = 2.01–2.2), titanium dioxide (*n*_1_ = 2.49), and tantalum dioxide (*n*_1_ = 2.3) for the first high index cladding and silicon dioxide (*n*_2_ = 1.46) for low index cladding, as they provide proper index contrast, for water core waveguides, and simple fabrication by means of silicon compatible process.

By combining these intrinsic properties along with an accurate design strategy, ultra-low loss, single mode linearly polarized ARROWs operation can be accomplished and exploited to realize high-performance interferometric devices.

### 2.2. 2-D Liquid ARROW

**Figure 2:**
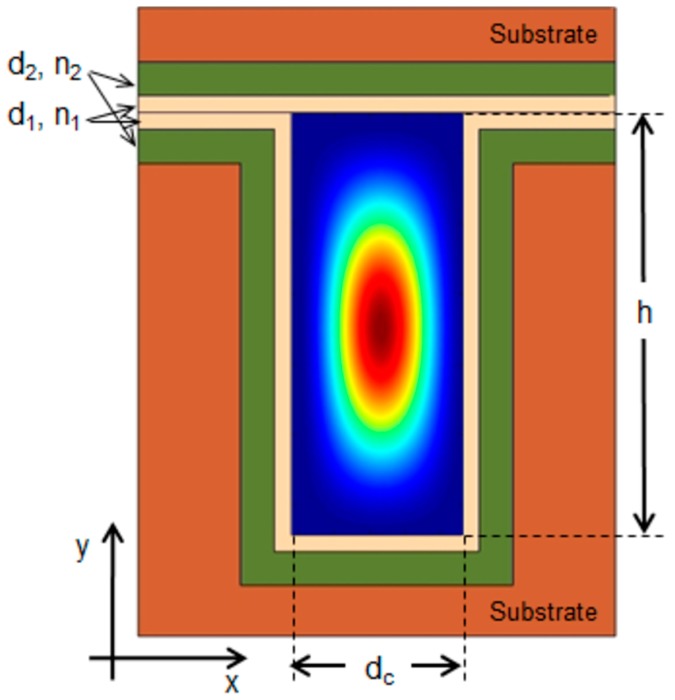
Cross-section of a 2-D liquid ARROW waveguide with simulated power distribution of the fundamental mode.

In a practical ARROW waveguide, the core must be completely surrounded by the cladding layers to accomplish light-confinement in both lateral and vertical direction. One possible ARROW structure for both vertical and horizontal confinement is illustrated in Figure 2.

**Figure 3:**
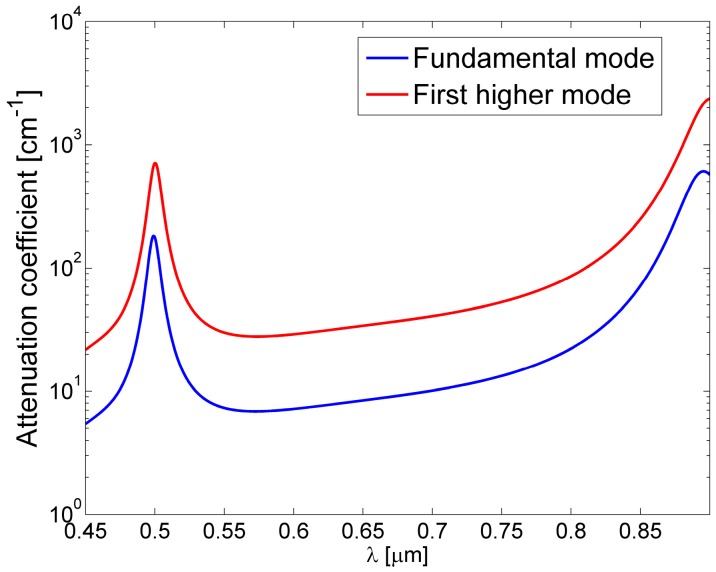
Attenuation coefficient of the fundamental mode (*E_y_*^0^) and the first order mode (*E_y_*^12^) *versus* the wavelength in water-core ARROW waveguide with *d*_1_ = 290 nm, *d*_2_ = 260 nm, *d_c_* = 5 µm and *h* = 10 µm (Figure 2).

A simple estimation of the attenuation coefficients in 2-D ARROW waveguides can be performed by using the one dimensional analytical model [11,15]. In a paraxial approximation the problem of the 2-D ARROW confinement can be separated into two 1-D problem for the lateral and transverse direction, in this way the evaluation of the total losses can be obtained from the addition of the resulting 1-D losses. In Figure 3 the losses for the fundamental (blue line) and first order mode (red line) *versus* wavelength calculated by using approximated analytical model, are shown. Hence, as anticipated, for a fixed core dimension higher order modes attenuate faster than the fundamental mode. From the figure the typical transmitted spectrum of an ARROW waveguide can also be foreseen. It is characterized by a broad spectral band, corresponding to the range of wavelengths which experience destructive interference in the claddings. The wavelength dependence of the propagation losses could be used to tailor the ARROW spectral response in sensing applications, *i.e.*, to filter out unwanted optical wavelengths from reaching the detector.

In 2-D ARROW design, the influence of the input polarization on waveguide losses should be taken carefully into account. If incident light is polarized along the *y*-axis (Figure 2) it is reflected upon lateral cladding as TE-polarized light whereas it is TM-polarized with respect to the vertical ARROW confinement. Hence, TE-polarized input light along *x*-axis will also contribute to the total losses as a TM polarization with respect to *y*-axis. By considering the polarization dependence of ARROW losses, these considerations suggest that the polarization of the propagating modes can be selected by suitably designing the core shape. If one of the core dimension is set wider (*i.e.*, a rectangular core shape), modes polarized along the longer side experience lower TM propagation losses with respect to the modes polarized along the crossing direction, *i.e.*, the longer core side identifies the direction of the supported polarization.

More accurate loss evaluation requires 2-D modes numerical analysis. A first detailed numerical investigation of 2-D hollow core ARROWs for sensing application is reported in [6]. The authors simulated ARROWs propagation by using finite element method (FEM) numerical analysis. The propagation characteristics of an ARROW with a hexagonal core cross section have been studied for different diameters of the core, filled with air or liquid substances. The authors also investigated the attenuation losses characteristics of the fundamental mode *versus* the detuning of the thickness of the first and second cladding layer from its antiresonant value. As it was found for solid ARROW [13], their study confirms that the attenuation losses are almost unaffected by the second layer thickness changes in a range of ±30 nm, whereas it is very sensitive to first cladding thickness, increasing very rapidly for thickness detuning of about ±10 nm. These results provide very useful rules for waveguides fabrication. 2-D numerical analysis of ARROWs modes characteristics have been also performed by using finite difference methods (FDM) [16,17].

## 3. Fabrication and Characterization

Currently, ARROWs are fabricated with two main different approaches: by bulk micromachining process or by surface micromachining process. In the first case, the ARROW is constituted by two halves of silicon substrate with the core etched in one of the halve part. The two processed silicon substrates are finally bonded after cladding depositions on both halves [17,18]. In the second case, the core is obtained by depositing and patterning a sacrificial material on a single silicon substrate in between top and bottom claddings. The sacrificial material is removed by etching at the end of process [19,20]. In both processes cladding layers are deposited using deposition techniques like plasma enhanced chemical vapor deposition (PECVD), low pressure chemical vapor deposition (LPCVD) or atomic layer deposition (ALD).

Both fabrication processes have some advantages and disadvantages. Bulk micromachining is a faster process but it needs a non-standard wafer bonding between high index materials like silicon nitride or titanium dioxide. However it enables a flexible design, as the top substrate can be substituted, allowing for the implementation of hybrid configuration, as it will be illustrated in the following. On the other hand, sacrificial etching of long narrow micro channels is very time consuming and it requires controlled manufacturing in order to achieve 100% fabrication yields [20]. Nevertheless, this approach allows a simple monolithic integration with solid core exciting/collecting waveguides. As it will be showed in the following, in both cases ARROW waveguides have been successfully produced and applied to fabricate very high performance and promising optofluidic devices for sensing applications.

**Figure 4:**
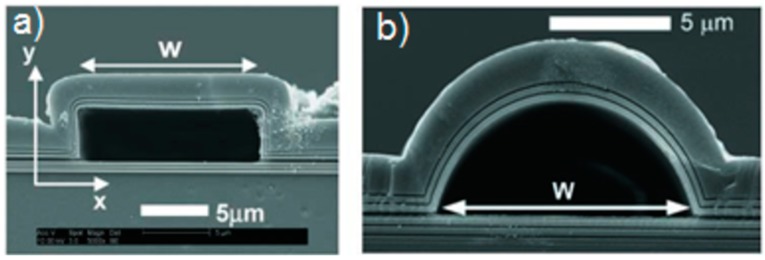
Scanning electron microscope (SEM) images of (**a**) hollow-core ARROWs with rectangular and (**b**) arch-shaped cross sections fabricated by surface micromachining process. Reprinted with permission from [27]. Copyright (2005) OSA.

**Figure 5:**
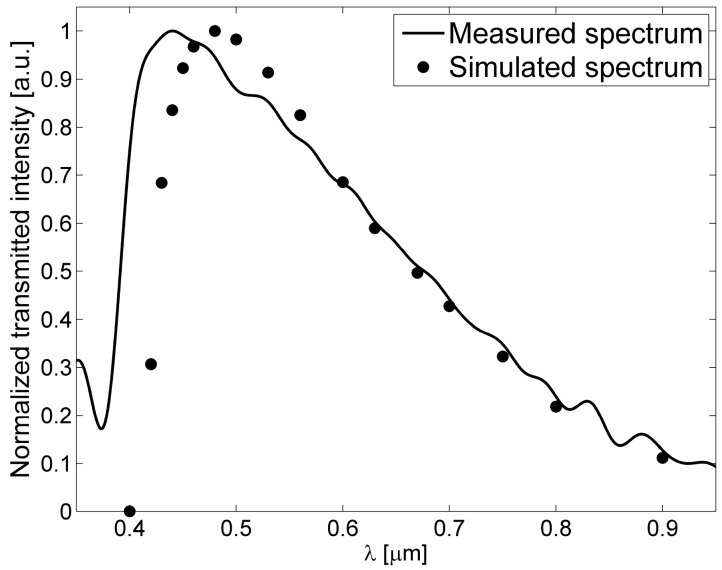
Measured and simulated transmitted spectrum from a single mode ARROW waveguide fabricated by ALD with *d*_1_ = 75.4 nm (TiO_2_), *d*_2_ = 262 nm (SiO_2_), *d_c_* = 10 μm, *h* = 5 µm, *n_c_* = 1.32 (methanol-filled core).

Single-mode ARROWs with hollow core have been firstly demonstrated by Yin *et al.* [21]. Hollow core dimensions were 3.5 µm by 12 µm, obtained by sacrificial etching of SU-8 (Figure 4a). For low-loss propagation, four antiresonant claddings were fabricated by alternating silicon oxide and nitride layer depositions on a silicon substrate using PECVD. The waveguide was designed to operate at the wavelength of 785 nm and with *n_c_* = 1. The authors demonstrated single-mode operation with propagation loss as low as 6.5 cm^−1^. The same waveguide was also characterized with a liquid-filled core (*n_c_* = 1.33), showing waveguide attenuation coefficient of 2.4 cm^−1^ at λ = 633 nm [19]. Successive improvements in the fabrication process have been proposed by Yin *et al*. [22] to reduce the waveguide losses as low as 2.6 cm^−1^ for 15 × 3.5 μm^2^ core dimensions (*n_c_* = 1). In this work the authors also discussed the effect on the waveguides losses of different growing rate over the horizontal and vertical core walls, which is intrinsic in the PECVD process. This effect causes that the vertical and horizontal layers do not simultaneously fulfill the antiresonance condition, deteriorating the waveguide performances. Even if this effect can be compensated by appropriate layer design [23], it can lead to non-optimal waveguide losses. In the work of Testa *et al.* [24] the ALD process has been proposed to overcome these limitations. ARROW waveguide was fabricated by using bulk micromachining, starting with the realization of the microchannels by etching 5 µm in the silicon wafer. Alternating layer of silicon dioxide and titanium dioxide were deposited on the core sidewalls by using LPCVD and ALD, respectively. One of the advantages of the ALD technique is the possibility to deposit very thin cladding layer (sub-100 nm) with an excellent conformality, reproducibility, and with a high precision over the resulting thickness. The waveguides were designed to operate on single mode at λ = 635 nm with a water filled core (*n_c_* = 1.33). The fundamental mode loss of 5.22 cm^−1^ was obtained with only two antiresonant layers. In Figure 5 the measured and simulated transmitted spectra of a 1.5 cm long ARROW waveguide fabricated by ALD are shown. Simulations have been performed by using by using a commercial software (FIMMWAVE, Version 5.2, © Photon Design) and FDM method. In the simulation model we have taken into account the wavelength dependence of the materials composing the waveguiding structure and the liquid filling the core (methanol). As Figure 5 shows, there is a discrepancy between the simulated and measured spectrum at low wavelength (below 400 nm). This discrepancy is due to the difficulty to obtain an accurate model for the core and the cladding layers refractive indexes (both real and imaginary parts) at wavelengths lower than 400 nm. In particular, as it can be noticed in figure, the spectral region at around 370 nm corresponds to the resonance condition in the cladding layers. As it is demonstrated in the [25], the wavelength position of the minimum in the transmitted spectrum (resonance in the claddings) is greatly influenced by the core and cladding refractive indexes.

Single-mode ARROWs with arc-shaped core were also demonstrated [26] (Figure 4b). The authors fabricated such structures by sacrificial etching and demonstrated their ability to overcome the mentioned limitation of the PECVD process. Further important advantages compared with rectangular ARROWs are the higher mechanical stability and the smoother core walls. Optical characterization of such structures with four antiresonant claddings was also carried out [27]. A low attenuation coefficient of 0.26 cm^−1^ was obtained at λ = 633 nm with a high refractive index liquid core (ethylene glycol, *n_c_* = 1.43).

Single mode hybrid silicon-polymer ARROW (h-ARROW) waveguide has been achieved by Testa *et al.* [28]. The authors exploited the advantage of bulk micromachining to separately process two silicon wafers, which were then bonded at the end of the cladding deposition. H-ARROW was obtained by substituting the upper silicon part with a single polydimethylsiloxane (PDMS) layer. Slightly increased optical losses were expected and measured due to the weaker optical confinement at PDMS-core interface compared with the stronger confinement enabled by antiresonant claddings. However, thanks to a proper design, this effect was minimized and fundamental mode loss of 6.18 cm^−1^ at λ = 633 nm (*n_c_* = 1.33) was obtained. A detailed study of h-ARROW confinement and loss improvement is reported in [16]. This approach circumvents the need of non-standard wafer bonding step between high index materials like silicon nitride or titanium dioxide, which is substituted by a very simple temporary or permanent bonding between silicon nitride and PDMS.

Single-mode liquid ARROWs with high performance have been demonstrated by using the two above-mentioned micromachining processes. Surface micromachining is very promising as it enables monolithic integration with exciting and collecting waveguides. As it can be envisioned, other components can be fabricated and integrated on the same platform in order to add further interesting functionalities for complete lab-on-a-chip realization. Bulk micromachining promises very high flexibility enabled by hybrid approaches. Polymer materials exhibit very favorable properties for both photonic and microfluidic technologies; moreover, thanks to the related inexpensive fabrication procedures, they show a great potential for economic mass production of lab-on-a-chip devices.

## 4. ARROW-Based Devices and Applications

Optofluidic waveguides have been demonstrated as very promising candidates for high performance optical microsensors [3,5]. Liquid ARROWs play a prominent role in this context, enabling planar integrated fabrication approaches based on the currently available and well-established silicon compatible technology.

Currently, the optical detection methods mainly applied in optical microsensors includes spectroscopic techniques like absorption, fluorescence, and Raman scattering. These approaches have demonstrated to be very effective for highly selective and sensitive detection of molecules down to the single molecule level. Additionally, the detection based on refractive index changes is widely used and have demonstrated a great potential for label-free detection of biomolecules with very high sensitivity.

In the following, we describe optofluidic design approaches for optical microsensors realized with liquid ARROWs.

### 4.1. Liquid ARROW Waveguides as Optofluidic Sensors

The strong light matter interaction occurring in the liquid core of an ARROW waveguide has been exploited to realize simple but effective and sensitive microfluidic sensors.

In the work of Campopiano *et al.* [25] the first demonstration of an integrated optical bulk refractometer based on multimode liquid ARROW has been proposed. For fluid injection, inlet, and outlet openings were fabricated in the silicon substrate. A multimode ARROW with large core section of 130 × 130 µm^2^ was used. The exciting and collecting optical fibers were inserted directly into the core, resulting in a highly compact and robust self-aligned configuration. Very notably, the waveguide, itself, constitutes the sensor. The sensing principle was based on the intrinsic property of the ARROW: the shift of the minimum of the transmitted spectrum upon core refractive index changes. Good bulk sensitivity of 555 nm/RIU (RIU: refractive index unit) and linear response, with a limit of detection (LOD) of 9 × 10^−4^ RIU has been demonstrated. The same configuration has been also used to realize an integrated long path absorbance cell for colorimetric detection of specific protein in water solution [17]. The authors used a Bradford assay for colorimetric determination of bovine serum albumin, showing an LOD of about 1 µg/mL with a sample volume of only 0.34 nL and a 15 mm-long-cell. This example was the first experimental demonstration of the sensing capability of a simple straight liquid core ARROW.

Multimode liquid ARROWs have also been employed to realize a flow cytometer for the analysis of fluorescently-labeled human T leukemia cells (Jurkat) [29]. The excitation light for cells interrogation propagated in the optofluidic channel thanks to the ARROW confinement, resulting in a highly compact and efficient optical interrogation scheme. The cells stream was hydrodynamically focused in the center of the channel where two optical fibers were arranged orthogonally for collecting the emitted fluorescent signals.

However, multimode ARROW with large core section area is not preferred to reach ultra-high sensitivity due to the large sample volume that typically causes strong background signal. This objective has been met by using single mode liquid ARROW. Thanks to the core section of a few microns, they provide a very small excitation volume, which is attractive for detection down to single molecule sensitivity.

In the work of Yin *et al.* [23], a liquid ARROW with picoliter core volume has been optimized and applied for highly efficient fluorescence detection of dye molecules, demonstrating an LOD corresponding to 490 molecules in the core. The potential for single molecule sensitivity by surface-enhanced Raman scattering (SERS) detection has been also demonstrated by Measor *et al.* [30] using a simple straight single mode ARROW.

The sensors above described can be considered as the first generation of ARROW-based optofluidic chips. However, more advances can originate from the use of optofluidic approaches to realize multifunctional photonic device and microfluidic elements on the same chip, as it will be illustrated in the following.

### 4.2. Interferometric Optofluidic Devices for Sensing Applications

Multimode and single-mode liquid ARROWs have been demonstrated as very promising tools for sensing applications because of their high compactness, sensitivity, and the inherit ability to transport the light and the analyte in the same physical channel. The potential for the realization of various functional optical elements on chip makes ARROW a suitable candidate for integration into compact photonic devices. Additionally, simply using the waveguide itself as optofluidic sensor, more sophisticated device architectures can, in principle, be realized by using liquid core ARROWs. In particular, the demonstration of single-mode ARROWs has provided the opportunity to implement optical sensors which make use of interferometric phenomena. For instance multimode interference (MMI) splitters based on liquid ARROWs have been demonstrated [31] and implemented in on-chip optofluidic interferometers [32]. Moreover, curved ARROW sections can be used for the realization of optical devices comprising light splitters or combiners, as it occurs in integrated optical interferometers. Bend waveguides based on ARROWs have been modeled by using 1-D FDM method [16] and fabricated in ring resonator geometry [33].

Planar optical integrated interferometric devices are very attractive for sensing applications as they combine high sensitivity with compactness. Typically, these devices are based on conventional solid core waveguides and exploit evanescent sensing to detect the presence of analytes in fluid sample [34,35,36]. Recently, innovative optofluidic architectures have been proposed for these devices to simplify fluids sampling and manipulation via microfluidic integration [4]. Very interesting optofluidic architectures for interferometric devices have been proposed based on droplet microfluidics [37] and fluid-air interface (meniscus) [38,39]. Several integrated optofluidic interferometric devices have been demonstrated based on liquid ARROWs, like wavelength division multiplexing (WDM) using MMI [40], ring resonators [41], and Mach–Zehnder interferometers (MZIs) [32,42].

**Figure 6:**
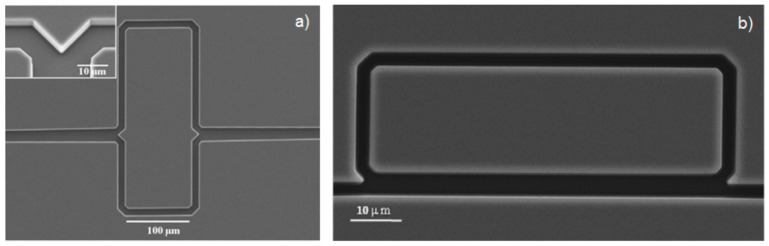
(**a**) SEM image of an integrated MZI based on liquid ARROWs. Reprinted with permission from [32]. Copyright (2010) OSA. (**b**) SEM image of an integrated optofluidic ring resonator based on liquid ARROWs. Reprinted with permission from [41]. Copyright (2010) AIP.

Optofluidic schemes for integrated MZIs in which a microfluidic channel is inserted along the sensing arm have been fabricated to allow the direct coupling of the light with sample fluid [43,44]. This design strategy enables a higher sensitivity thanks to the almost complete overlap of the mode power with the sample fluid in the sensing region. However, in these cases the interaction length is limited by the width of the microfluidic channel. Moreover, the high insertion losses at solid/liquid interface in the inserting region can cause poor device performances. With the advantages offered by liquid ARROW, a planar integrated optofluidic MZI entirely shaped by liquid microchannels has been fabricated [42]. The design consists of two asymmetric single-mode branches split from a Y-Branch and recombined by an inverse Y-Branch in single mode waveguide for interfering. Further improvement in sensor design has been proposed in the work of Testa *et al.* [32] to minimize the power unbalancing between arms and to increase the visibility of the interferometer (Figure 6a). The device features large RI tunability, typical of the optofluidic microsystems, as the guiding condition is satisfied for quite a large RI range *n_c_* = 1.32 ÷ 1.45, that corresponds to the detection range of the sensing device. RI limit of detection (LOD) of Δ*n* = 1.6 × 10^−5^ RIU has been estimated, which compares well with results reported for other optofluidic MZI for label-free sensing of liquids [43,45]. However, while evanescent based-MZIs have been demonstrated as extremely sensitive in direct and real-time measurement of biomolecular interactions [46], optofluidic MZIs have been mostly applied for RI volume sensing of liquids due to the enhanced bulk interaction occurring in the optofluidic channel. Detection of single viruses and the discrimination between different kinds of virus types in a sample mixture have been demonstrated using an optofluidic heterodyne interferometer employing an out-of-plane detection/interrogation optical scheme [47].

Optofluidic ring resonators (ORRs) have also been recently proposed. Compared with MZI-based sensors that exploit limited sensing lengths, optical resonators benefit from the repetitive interaction between the light and the sample, achieved thanks to the resonant recirculation of light in the cavity. Thanks to this property, highly compact devices can be fabricated with very high sensing capability. Several architectures for optofluidic resonators have been demonstrated which incorporate fluidic capabilities into the photonic structure, such as the capillary-based optofluidic ring resonators (CORRs) [48,49,50]. The CORR is a microfluidic capillary that supports resonant optical modes (the so-called whispering gallery modes (WGMs)) circulating in the capillary wall with the evanescent field extending into the fluid.

Liquid core ARROW has been successfully employed as a basic element to form an integrated ORR (Figure 6b) [41]. A first generation of ARROW-based ORR has been fabricated with a moderate quality factor (Q-factor) on the order of 10^3^, mainly due to the high optical losses of the curved sections. Advances in the ORR performances were experimentally obtained by performing an accurate optimization on the initial structure design (first-generation ORR), including those concerning the optofluidic level of integration [16]. A planar ORR based on liquid ARROWs with a Q-factor of about 10^4^ has been demonstrated by applying the optimization procedure [33]. The proposed approach retained the merit of high level of optofluidic integration in a planar configuration. The strong light-matter interaction occurring in the liquid core confers high bulk RI sensitivity to the ORR, experimentally estimated as about 700 nm/RIU. This value is larger than that obtained by most of the other optofluidic ring resonator sensors like slot- and capillary-based ORRs [50,51,52]. In order to be applied for biomolecules detection, the surface sensing performances of the device have been simulated, showing very promising capabilities [16]. In comparison, the CORRs have demonstrated very good surface sensing. Biotin molecules captured on the wall surface have been detected with a detection limit of approximately 1.6 pg/mm^2^ [51]. Breast cancer biomarkers (CA15-3) have been also detected at a concentration of 1–200 units/mL in phosphate-buffered saline buffer and 20–2500 units/mL in fetal calf serum [53]. However, tapered optical fibers are typically used to excite WGMs, which hinders the integration of CORRs onto a compact lab-on-chip platform.

### 4.3. Planar Integrated ARROW Platform

One of the most relevant problems associated with the chip-scale integration of liquid core waveguides concerns the way light is collected from the open-ended liquid core. An optimized transmission between liquid core waveguides and off-chip components is necessary to increase the overall optical throughput. In particular, the ability to efficiently collect the light is crucial for achieving single-particle detection. Not surprisingly, the first demonstration of single molecule sensitivity using an open-ended liquid ARROW required bulk optical components, such as a high numerical aperture objective lens [23,30].

Technological solutions to provide on chip integration of intersecting microfluidic channels and optical waveguides are an object of many works in the literature [54,55,56,57,58]. In addition to the obvious implications to enable miniaturization and simplify the alignment procedure, on-chip integrated waveguides offer two further remarkable advantages. First, the possibility to improve the optical coupling at the waveguide-fluid interface that in turn improves the signal to noise ratio and the sensitivity of detection. Second, they can be suitably placed on-chip in order to illuminate only specific points along the liquid core waveguide, thus enabling highly spatially-resolved optical detection on a sub-picoliter excitation volume. Using surface micromachining technology, the first step towards the realization of an integrated platform based on liquid ARROW has been proposed in the work of Yin *et al.* [59]. The authors demonstrated an important advance in sensor design, obtained by integrating solid core with liquid core ARROW in a full planar geometry. Parallel arrays of solid core waveguides were arranged orthogonally with liquid waveguides for fluorescence measurements on sub-picoliter volumes. The proposed approaches resulted in a network of intersecting waveguides for specific excitation of well-defined locations along the liquid core waveguide. Thanks to this geometry, strong pump suppression and sensor sensitivity down to a single molecule have been achieved. Using a similar approach, a solid core waveguide aligned with the liquid waveguide has been integrated on an ARROW platform for surface-enhanced Raman spectroscopy (SERS) detection with molecular specificity [60]. In this configuration the solid waveguide was collinear with the liquid waveguide and it was used to excite the entire liquid volume. Moreover, in order to prevent liquid evaporation during measurements, a further remarkable improvement has been proposed, consisting in an on-chip reservoir of 10 µL volume for continuous piping of fluid solution. This has been the first implementation of a reliable optofluidic tool on an ARROW sensing platform. Using this platform, the authors detected the minimum active R6G concentration of 30 nM, corresponding to measuring approximately 4 × 10^5^ molecules in the ARROW volume of 44 pL. Other very interesting optofluidic devices for SERS detection have been demonstrated with better limits of detections, but generally requiring out-of-plane bulk instruments for the measurements [61,62].

Step-by-step, successive improvements of the optical and sensing performances of the liquid ARROWs have also been made from a fabrication point of view [63,64,65,66]. In [65] tantalum oxide and low temperature-deposited silicon nitride were proposed to reduce the background contribution of native photoluminescence of the cladding material in fluorescence measurements. With this arrangement the authors demonstrated a signal-to-noise-ratio (SNR) improvement by a factor of 12 in fluorescent nanoparticle detection compared to conventional silicon nitride-based ARROWs. Optimization of interface transmission between the liquid and solid ARROWs has also been performed to achieve high sensitivity in spectroscopic measurements [66,67].

**Figure 7:**
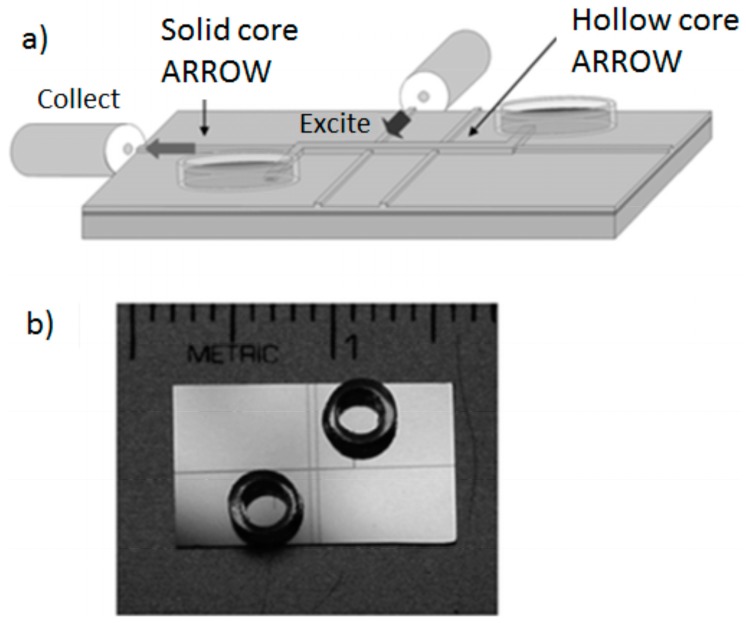
Planar optofluidic chip. (**a**) Schematic view of the integrated optofluidic chip showing hollow- and solid-core ARROW waveguides and (**b**) photograph of fabricated chip. Reprinted with permission from [68]. Copyright (2007) RSC Publishing.

A chip-scale integrated planar sensing system that combines optical and fluidic functions is required to address the challenge of high sensitivity and compactness. By using a liquid ARROW integrated with both orthogonal and aligned solid waveguides, together with sample reservoirs, the first planar optofluidic chip for single particle detection and analysis has been demonstrated [68]. The chip has been fabricated by using surface micromachining planar silicon technology. In this design, solid waveguides are not only used to precisely excite a sub-picoliter liquid volume but also to collect and deliver light from liquid ARROW towards off-chip fibers (Figure 7). The advantages offered by this design of providing multiple and spatially separated collection paths have enabled the implementation of advanced multi-color spectroscopy techniques on chip, such as fluorescence cross-correlation spectroscopy (FCCS) for the detection and discrimination between nanobeads labeled with different fluorescent dyes [69]. The chip has been successfully employed to detect a single bacteriophage Qβ virus without the need for particle immobilization [70]. The achieved sensitivity is approaching detection of virus on single level and is comparable to sensitivity reported with other microfluidic chips [71,72,73] and other integrated techniques, such as evanescent waveguide sensors [74,75,76], electrical nanowire arrays [77], and nanoelectromechanical cantilevers [78]. More recently, a solid-state nanopore has been integrated in the optofluidic chip. The nanopore ensures controlled delivery of individual bioparticles into the ARROW optofluidic channels. The bioparticle detection involved both electrical and optical measurements. Using this arrangement, the authors demonstrated single-particle detection of a fluorescently-labeled influenza virus and λ-DNA [79,80]. Optical detection techniques that complement the electrical measurements are well-known as very powerful, high-sensitive tools for single-particle detection using nanopore sensing [81]. However, the proposed optofluidic chip presented the advantage of multi-level integration of fluidic, optical, and electrical control on a single chip.

Manipulation of bio-particles in a fluid sample is of great interest in the field of optofluidics as it enables analysis of biomolecules at a single level [82]. Basically, by using the chip design proposed in [68], very interesting operations have been demonstrated based on an all-optical particle control via the integrated waveguides, *i.e.*, the trapping and sorting of the particles in a fluid flow [83,84,85,86]. The planar nature of the optofluidic chip suggest that this technique could be applied for sample processing of a liquid with suspended bioparticles (viruses, bacteria, cells, and other microorganisms) for on-chip single particles for optical sensing and analysis. The authors exploited the combination of optical trapping and fluorescence detection of single microorganisms by evaluating the photobleaching dynamics of stained DNA in *Escherichia coli* bacteria [85].

In striving for miniaturization, several efforts have focused on the integration of various optical components on chip, such as slits and lenses [87,88], mirrors [89], filters [90,91], *etc*. Spectral filters are commonly used to reject pump contribution and distinguish the sample signal from background noise. Integration of optical filters on planar platforms can greatly support device miniaturization for high sensitivity in spectroscopic techniques, avoiding the use of bulky optical components [90]. In an ARROW waveguide, wavelength filtering can be achieved by exploiting the inherent spectral dependence of the confinement mechanism [11]. In the work of Philips *et al.* [92], an integrated notch filter has been implemented on a planar ARROW optofluidic chip for spectroscopic applications. The interference claddings of the collection solid waveguides have been suitably designed in order to confine the analyte signal wavelengths while rejecting the excitation light. In the work of Measor *et al.* [93] a similar approach has been used to tailor the spectral response of both liquid and solid core ARROWs. The authors demonstrated the ability to simultaneously reject the pump light while preserving low loss propagation of the analyte signal in Forster resonance energy transfer (FRET) detection of doubly-labeled oligonucleotides. High extinction of 37 dB was shown with a 4 mm long optofluidic filter. The estimated LOD of 15 nM is comparable with other integrated approaches [91].

In the examples presented so far, although the high level of optofluidic integration, the microfluidic functionality is essentially restricted to the ARROW ability to host fluids. On-chip devices for complete bio-medical and chemical analysis, however, often require more sophisticated microfluidic functions, *i.e.*, to accurately mix fluids and control their flow rates. Optofluidics arises from the need to move these functionalities on-chip without sacrificing the optical part. Actually, optofluidics attempts to find design solutions to suitably merge fluidic and photonic elements and improve the overall device functionalities. It is also becoming even more recognized that microfluidic systems can be more easily fabricated using polymer materials and related fabrication technologies. This choice benefits from the reduced cost and simpler fabrication procedures, which could promote larger-scale diffusion. On the basis of these concerns, ARROW-based optofluidic chips that make use of a hybrid technology to integrate microfluidic system have been recently developed by exploiting both surface and bulk micromachining fabrication procedures.

**Figure 8:**
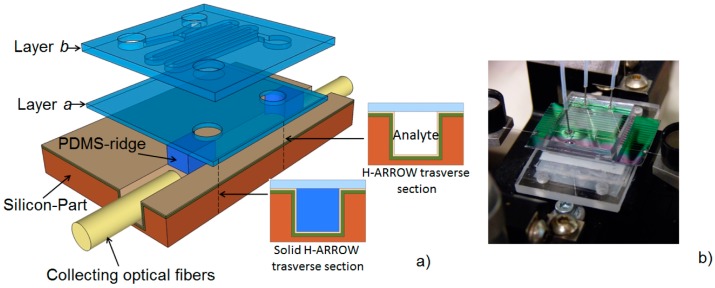
(**a**) Schematic of the proposed hybrid ARROW optofluidic platform with transverse section of liquid h-ARROW and solid h-ARROW; (**b**) Photograph of fabricated chip. Reprinted with permission from [95]. Copyright (2014) OSA.

Based on bulk micromachining process, a hybrid ARROW optofluidic platform has been developed using the combination of PDMS and silicon-based materials. PDMS is extensively used in microfluidic technology because of its optical transparency in the visible/UV region, the biocompatibility and the low-cost fabrication procedures, like soft lithography [94]. The opportunity of combining silicon and polymer technologies has been firstly demonstrated with the fabrication of a single-mode h-ARROW, as reported in the work of Testa *et al.* [28]. Starting from this vision, the optofluidic platform has been assembled in a modular structure, with the upper polymer parts having both optical and microfluidic functions [95] (Figure 8). In particular, by moving from the bottom to the top of the structure, in the first PDMS layer (layer a) hybrid solid-core ARROWs have been integrated to deliver the optical signal towards the collection optical fibers. They also sealed the open-ended channels, preventing liquid evaporation. Merely fluid handling functionalities were restricted to the second PDMS layer (layer b), where a microfluidic mixer was incorporated. Such layer can be easily replaced with another, in order to adapt the microfluidic system to specific sensing applications. This confers to the platform high design flexibility. A multimode h-ARROW was used in order to allow self-aligned positioning of off-chip fibers with solid waveguides through the optofluidic channels. The sensing performance of the device was tested by performing fluorescence measurements at different dye concentrations, controlled by means of the integrated micromixer; an LOD of 2.5 nM was demonstrated. Even if this value is high as compared with the current state of the art of fluorescence measurement using evanescent waveguide sensors [96], the proposed modular approach for chip assembling into a compact and multifunctional format is very promising and offers prospects of an increased flexibility. For comparison, a modular approach for slot biochemical sensors have also been recently proposed in the work of Carlborg *et al.* [52] An array of optical slot-waveguide ring resonator sensors has been packaged into a chip incorporating microfluidic sample handling, with a microfluidic layer assembled in a compact cartridge. The authors demonstrated a volume refractive index detection limit of 5 × 10^−6^ RIU and surface mass detection limit of 0.9 pg/mm^2^.

**Figure 9:**
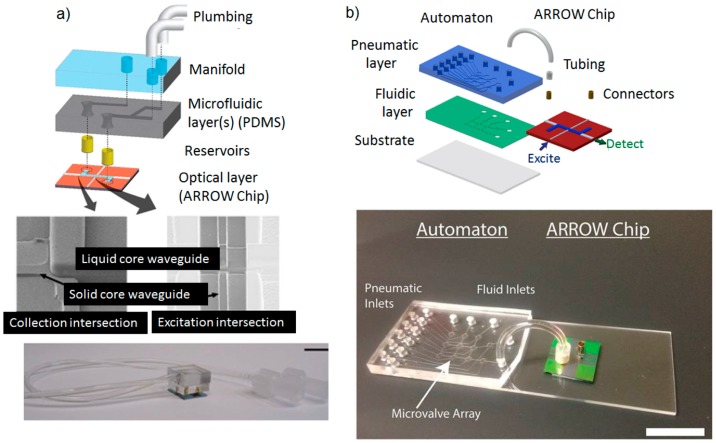
(**a**) Expanded view of PDMS integration with an ARROW optofluidic chip. Pictures are SEM images of optical intersections. Reprinted with permission from [97]. Copyright (2013) RSC Publishing. (**b**) Schematic of hybrid integration of automaton and ARROW chips. Reprinted with permission from [98]. Copyright (2014) AIP Publishing.

A hybrid ARROW optofluidic platform has been also proposed in the work of Parks *et al.* [97]. In this configuration, the fluidic and optical chips were two separate entities, connected via metal reservoirs as shown in Figure 9a. The fluidic layer was fabricated in PDMS by soft lithography, being aligned but not in contact with the optical layer. This allows great design reconfigurability, as the PDMS layer can be removed and replaced without losing the alignment of the fluidic reservoirs with the optical chip. Microfluidic capabilities of fluidic mixing, distribution, and filtering have been demonstrated in combination with single-molecule fluorescent detection of dye-labeled λ-DNA. Using a similar approach for microfluidic integration, a more sophisticated fluid handling system capable of advanced microfluidic functions has been integrated with the optofluidic chip (Figure 9b). In particular, an actively programmable microfluidic layer made of PDMS was obtained by employing an interconnected array of nanoliter microvalves. Precise sample handling functions such as mixing, splitting, delivering, and storing were implemented for molecular diagnosis of viral nucleic acids from complex solutions [98], and for amplification-free detection and quantification of Ebola virus on clinical samples [99]. By using the advantages of the integrated PDMS-based microfluidic chip (automaton) to process the fluid sample, the authors demonstrated a LOD of 0.2 pfu/mL, comparable with other amplification-free methods like PCR analysis [100] and more than four orders of magnitude lower than other chip-based approaches [101].

## 5. Conclusions

The discussion in this review demonstrates that the liquid core ARROW is a valuable tool for optofluidic integration. ARROWs have been successfully fabricated using current silicon technologies, showing very high optical performances. With core sections of a few microns, these waveguides can contain ultra-small fluid volumes of nanoliters to a few picoliters and simultaneously analyze it down to the single-molecule detection. As demonstrated by discussed examples, ARROWs have the capability to enhance the sensitivity of detection in several optical methods of analysis like fluorescence, Raman spectroscopy, and refractometry. Using ARROWs, complex interferometric sensing devices like a Mach–Zehnder interferometer or ring resonators have been designed and successfully tested.

Integrated optofluidic platforms have been demonstrated by combining solid core with liquid core ARROWs and microfluidic devices in several planar configurations. This represents a significant step forward from the previous version of an ARROW platform, opening a path towards an effective realization of chip-scale integrated system. The integration of planar optical components with microfluidic tools at the chip-scale represents the future trend of sensing systems for point-of care diagnosis or *in situ* monitoring. Increasing the synergy between these technologies can lead to highly reliable and portable systems.

By using ARROW optofluidic platforms, several advanced sensing methods have been successfully implemented for the manipulation, detection and analysis of single bioparticles directly on chip. The integration of microfluidic systems capable of advanced operations into an ARROW optofluidic chip has been the topic of intense investigation in recent years. Novel solutions have been proposed that are based on hybrid silicon/polymer technologies. The use of polymer to fabricate the microfluidic system has the potential to address the requirements of reduced fabrication cost and simplified manufacturing. The possibility to realize an optofluidic infrastructure of interconnected solid- and liquid-core waveguides on a planar silicon substrate and the hybrid integration of the ARROW platform has been demonstrated to be very attractive and creates a pathway for further integration of optoelectronic components towards a complete and self-contained chip integrated system. Based on the great improvements obtained in recent years, further progresses can be envisioned in fabrication methods that will improve the overall functionalities and promote commercial potential of ARROW-based optofluidic chip.
